# Strengthening Professional Collaboration and Expertise: Implementing and Sustaining a Hospital School Community of Practice

**DOI:** 10.5334/cie.165

**Published:** 2025-07-09

**Authors:** Miranda Field, Heather Lewis

**Affiliations:** 1 University of Regina, Canada; 2 Regina Public Schools, Canada

**Keywords:** community of practice, hospital schools, hospital teacher, professional development, collaboration

## Abstract

This practice-based intervention paper describes the design and implementation of a Community of Practice (CoP) intervention for hospital school professionals across three hospital sites in Saskatchewan, Canada. The intervention consisted of four structured sessions per academic year, two mandatory and two optional, offered during school division professional development days. Each session included facilitated dialogue, guest speakers, collaborative planning, and resource sharing tailored to the realities of hospital-based and inclusive education. Structured using Wenger’s theory of social learning and reported following the GREET (Guideline for Reporting Evidence-based practice educational interventions and Teaching) framework, the CoP was conducted from September 2016 through June 2021. It involved elementary and secondary teachers from hospital schools, complex needs programs, mainstream schools, and student teachers, totaling between 7 and 11 participants annually. The intervention aimed to address the unique professional development needs of hospital teachers, mitigate professional isolation, enhance interdisciplinary collaboration, and support evidence-informed practices tailored to students aged 5–17 with complex medical and mental health needs. Data were collected through attendance records, facilitator notes, participant feedback, and reflective forms. Analysis employed a thematic approach using deductive alignment with predefined learning objectives and inductive methods to identify themes. Results indicated the CoP effectively fostered relational trust, professional renewal, adaptable resource co-creation, and sustained engagement despite systemic and bureaucratic constraints. Knowledge translation efforts included podcast interviews, conference presentations, and field trips to showcase and disseminate the CoP model. Recommendations include integrating robust evaluation frameworks at the intervention outset. This intervention provides valuable insights for replicating CoP models, the intervention, within similar interdisciplinary education-healthcare contexts.

In Canada, numerous urban hospitals provide school support to students aged five to seventeen, ensuring continuity of education for children and youth facing acute or chronic medical conditions. (Calgary Board of Education, 2025, June 7; Saskatoon Public Schools, 2025, June 7; The Hospital for Sick Children, 2025, June 7). These students often face disruptions in academic engagement, social participation, and mental health due to hospitalization or ongoing treatment (Glang et al, 2018; Ratnapalan et al, 2009). Outcomes for these learners, when supported by appropriate interventions, can include improved academic performance, increased self-efficacy, and smoother reintegration into community or mainstream schools (Hopkins et al., 2014; St. Leger, 2014; Ratnapalan et al, 2009). However, the landscape of hospital education is increasingly complex, shaped by rising medical and mental health challenges among school-aged children (Lowe et al, 2024). In most provinces, hospital schools are considered a part of the public education system and fall under the legislative framework that governs provincial Ministries of Education.

These programs are typically funded by school divisions or boards and staffed by certified Canadian teachers, though delivery models and administrative structures vary across jurisdictions (Saskatchewan Ministry of Education, 2013). Hospital school teachers work in clinical settings but remain accountable to educational authorities, navigating a dual system that merges education and healthcare requirements. This structural positioning creates both opportunities and challenges for interdisciplinary collaboration and equitable service delivery. Hospital teachers are certified educators who provide instruction to students receiving care in medical settings, including acute care hospitals, rehabilitation centres, and mental health units. They play a critical role in maintaining academic continuity during hospitalization, adapting curriculum to accommodate a wide range of medical, emotional, and cognitive needs. Unlike traditional classroom teachers, hospital teachers navigate dynamic clinical environments, collaborate with multidisciplinary teams, and tailor their instruction to students whose health conditions may change daily. Their work requires flexibility, compassion, and an in-depth understanding of both educational and medical considerations, making their role distinct within the broader field of education.

Despite the essential role they play, hospital teachers remain underrepresented in broader educational research and policy. Research has documented the need for integrated supports that respond to the developmental, cognitive, and emotional needs of these students (Cave et al, 2020; Hopkins et al, 2014; St. Leger, 2014). However, professional development opportunities that address the unique realities of hospital school teachers remain scarce (Hen & Gilan-Shochat, 2022; McNamara, 2024). Hospital teachers often work in small teams or within multidisciplinary contexts, navigating rapidly shifting clinical environments where the role of education is frequently undervalued; the medical model does not always recognize the unique contributions hospital teachers make to the overall care and well-being of school-aged patients during admission (Benigno & Fante, 2020; Hen & Gilan-Shochat, 2022; Jiliberto & Zarate Alva, 2025; Malkowska-Szkutnik, 2021).

This intervention describes a Community of Practice (CoP) implemented across three hospital school sites and two school divisions in Saskatchewan, Canada which sought to address the unique professional development needs of hospital school teachers. The CoP was an intervention aimed at reducing professional isolation, enhancing instructional and transition planning strategies, and building a sustainable collaborative culture across health and education disciplines. Grounded in Wenger’s (1998) theory of social learning and aligned with the GREET checklist for reporting educational interventions (Phillips et al, 2016), this paper describes the implementation of a CoP and provides an overview of the intervention’s structure, delivery, and outcomes.

## Theoretical Overview of a Community of Practice

A CoP is a collaborative professional learning structure grounded in social constructivist and situated learning theories, where learning occurs through shared experience and mutual engagement (O’Brien & Battista, 2020; Terry et al, 2019; Wenger 1998). Wenger (1998) describes CoPs as groups with a shared domain of interest who build knowledge collectively through regular interaction. In educational contexts, CoPs support professional identity, reduce professional isolation, and enable co-construction of tools and practices tailored to specific teaching environments (Buckley et al, 2019; Cojorn & Sonsupap, 2024; McNamaro, 2024). For this intervention, the CoP provided a structured, yet flexible space for educators working in hospital schools to connect, reflect, and collaborate. The model emphasized active participation over passive delivery, with members contributing to agendas, leading discussions, and sharing resources in response to emergent needs. According to Giusti et al. (2017), CoPs are effective mechanisms for generating high-value knowledge through collective inquiry and lived experience. Kitto et al. (2018) further stress that authentic CoPs require distributed leadership and member-driven content. By bringing together professionals across geographic and disciplinary boundaries, this CoP created a sustainable learning culture that valued both experiential and formal knowledge. It cultivated relevance, trust, and shared accountability—principles essential for educators navigating the dual landscapes of healthcare and education.

## Design and Methodology

The CoP intervention was implemented for five academic years (September-June), 2016/2017 through 2020/2021, following a multi-phase design: (1) Planning and Engagement, (2) Implementation, and (3) Reflection and Sustainability each academic year. Sessions were delivered in-person at hospital schools with participation from out-of-city attendees supported virtually. During the COVID-19 pandemic, the CoP shifted fully to online delivery. Data were collected through attendance records, facilitator notes, participant feedback, and end-of-year reflections. A thematic analysis approach was used to analyze qualitative feedback related to participant experiences and alignment with learning objectives, and to identify emergent themes across multiple years of implementation. Each of these aspects will be described further in the sections below.

### Participants

Participants were recruited through existing hospital school networks and professional learning communities supported by the Saskatchewan Ministry of Education and school divisions. The facilitators of the CoP invited teachers through a direct email, phone call, or school-based outreach. The facilitators wrote in their recruitment call:

*“The purpose of this Community of Practice is to create a space where we can support one another in navigating the unique realities of hospital and complex needs education. Together, we will explore strategies to strengthen re-entry planning, trauma-informed practice, and the role of professional identity in sustaining this work. Whether you’re a hospital teacher, a complex needs educator, or someone supporting students through transitions, you are welcome to join.”*

Participants were elementary and secondary school teachers, teaching students aged five-seventeen, from hospital and mainstream (not hospital) schools, including those supporting students with complex needs. A hospital school teacher is a certified educator who provides academic instruction to students receiving medical or mental health care in hospital settings. These teachers support continuity of education during hospitalization, often adapting curriculum to meet individual health and learning needs within a multidisciplinary care team. A complex needs teacher is a certified educator who works within a school setting with students requiring intensive supports due to a combination of cognitive, behavioural, emotional, or physical challenges. These may include students with autism, developmental disabilities, significant anxiety, or aggressive behaviours, often within dedicated programs or classrooms designed to support individualized learning and regulation. A mainstream teacher is a certified educator who teaches in a general education classroom, often responsible for straight or split-grade classes within school settings. These teachers support a diverse range of learners and frequently reintegrate students returning from hospital or specialized programs into the school community. Mainstream classroom teachers were welcomed into the CoP, as they often support students upon their return to school following hospitalization or have a personal connection to the life of a student in the hospital. Students with lived-experience, educational psychologists, child life specialists, and allied health professionals contributed as guest speakers during specific sessions. Participation was voluntary, supported by school division supervisors, and integrated into school division professional development. Table 1 provides an overview of the participant groups who engaged in the CoP throughout its implementation.

**Table 1:** Overview of the Community of Practice Participants.


PARTICIPANT ROLE	YEAR 1	YEAR 2	YEAR 3	YEAR 4	YEAR 5

Hospital Teacher	3	5	6	5	4

Complex Needs Teacher	2	2	2	2	2

Mainstream Classroom Teacher	1	1	3	2	1

Student Teacher	1	0	0	1	0

Total Participants	7	8	11	10	7


### Context

The CoP was implemented in the province of Saskatchewan, Canada, within three hospital school settings located in two urban centres. These urban centers are home to Saskatchewan’s largest hospitals and serve a diverse student population from across the province. Hospital schools in Saskatchewan are staffed through public school divisions, funded by the provincial government, but operate within provincially funded public hospitals. The first site, an urban general hospital, served approximately 550 students annually across inpatient acute care pediatrics, inpatient adolescent mental health, and occasional outpatient pediatric services. The second site included an urban rehabilitation hospital and a home tutorial program, jointly supporting fewer than approximately 25 students per year. This site focused on inpatient pediatric respite as well as delivering homebound instruction in partnership with local school divisions. The third site was an urban children’s hospital serving a broad and complex caseload of approximately 550 students annually, including those in inpatient pediatric acute care, mental health programs, and extended homebound education placements. Students in these programs often experience significant disruptions in their educational trajectories due to long-term or recurring medical and mental health treatment. The CoP addressed not only clinical and educational challenges but also relational and systemic barriers between healthcare and education.

### Environment

CoP professional development sessions were initially held in person at hospital school sites, with out-of-city participants joining via video conferencing. These hybrid meetings allowed for both face-to-face collaboration and remote participation. During the COVID-19 pandemic, sessions transitioned to fully virtual delivery.

### Learning Objectives

The intervention addressed five core learning objectives:

1. To enhance instructional strategies for students with complex medical and mental health needs.
2. To share resources and tools to support continuity of learning.
3. To build trusting relationships and interprofessional collaboration.
4. To support professional identity and well-being of hospital schoolteachers.
5. To explore and leverage partnerships with community and health systems.

### Materials

Materials were carefully curated and adapted across the phases of the CoP and informed by participant feedback as well as key references in the field. A sample of materials included:

- Facilitator-developed templates and planning guides: Agenda templates, meeting facilitation prompts, and visioning tools such as the ‘community engagement spectrum’ and leadership inventory handouts (Center for Community Health and Development, 2019; Manitoba Education and Training, 2017).
- Visual tools and reflective practice aids: Identity mapping exercises, relational ecosystem drawings, story circle prompts, and wellness wheels adapted from youth voice and Indigenous-focused educational methods (Regan, 2010; Hart, 2002).
- Professional learning materials: Curated book excerpts (e.g., Truth and Reconciliation Commission of Canada, 2015), podcast episodes from “Trauma Informed Educators Network,” open-access articles (e.g., Atleo, 2011), and handouts on cultural safety and vicarious trauma (BC Provincial Outreach Program, 2020; Benigno et al, 2018; Curtain et al, 2019; Dwyer et al, 2012; KPJR Films, 2016; Ministry of Education British Columbia, 2016; National Child Traumatic Stress Network, 2008; Saskatchewan Advocate for Children and Youth, 2016; Souers & Hall, 2016; The Pennsylvania Child Welfare Resource Center, 2025, June 7).
- Transition and re-entry resources: Sample re-entry plans, care coordination templates, and visual communication boards for students returning to school post-treatment (Hopkins et al, 2014; Saskatchewan Ministry of Education, 2018; SickKids, 2016).
- Student-centered planning tools: Personal wellness tools, individualized learning plans, culturally responsive Individual Education Plan supports, and adapted student profiles reflecting diverse needs (Beads of Courage, 2019; Brownlie & King, 2011; Elam et al, 2019; Gutman & Irwin, 2020; National Association of Chronic Disease, 2025, June 7; Saskatchewan Teachers’ Federation, 2017).
- Crisis and grief supports: Resources on grief-responsive schooling, classroom protocols following medical emergencies, and peer support resources (Dyregrov, 2008; BC Children’s Hospital, 2020).
- Documentation and sharing tools: Collaborative Google Drive folder, editable planning templates, shared slide decks, and anonymized composite cases used for cross-site learning (Collaborative for Academic, Social, and Emotional Learning, 2015; Goosechase, 2019; Wenger et al, 2002).

## Implementation of the Intervention/Activities

### Educational Strategies

Educational strategies included structured discussion, peer-to-peer sharing, storytelling, guest speaker sessions, visual facilitation, collaborative inquiry, and off-site excursions. Strategies were selected based on adult learning principles (Dempsey et al, 2022; Jared et al, 2023; McCarthy et al, 2019).

### Instructors

The CoP was co-facilitated by two teachers with expertise in hospital school systems and professional development. The first two years of the CoP were facilitated by one teacher, the last three years were co-facilitated by two teachers. These teachers also served as consistent co-facilitators throughout three years of the intervention and were from the hosting school division. Facilitation duties rotated among CoP participants periodically to promote shared ownership. Guest presenters included pediatricians, Indigenous Elders, social workers, professional advocates, and students with lived experience.

### Program Delivery and Schedule

Meetings were held in alignment with school division-designated professional development days to ensure accessibility and institutional support. Each academic year included two mandatory sessions and two optional sessions between September and June, providing flexibility while maintaining structure for sustained engagement. Sessions were approximately 150 minutes and alternated between structured professional learning and open dialogue. Table 2 outlines a sample schedule from Year 3 of the CoP to illustrate the structure, timing, and content focus of a typical implementation year.

**Table 2:** Sample schedule Year 3 of the Community of Practice Activities (2018–2019).


DATE	DURATION	FOCUS TOPICS	KEY ACTIVITIES

Fall, 2018	2.5 hours (required)	Trauma Informed Practice, Attendance Matters: School Refusal and Re-Entry, Teacher Wellness	presentation and discussion, group brainstorm, make and take self-care plan

Winter, 2018	2.5 hours (optional)	Shared Lived Experience, Adolescent Psychiatry, Provincial Resources, Forward Planning	former student guest speaker, multi-disciplinary panel presentation with q & a, resource sharing using jigsaw method

Spring, 2019	1.5 hours (required) 1.0 hours (optional)	Student Advocacy, Children’s Rehabilitation Program, Forward Planning	review published report and discuss proposed actions in-context, on-site tour, electronic survey

Spring, 2019	Full day (optional) 5.0 hours travel 4.0 hours collaboration	New In-province Children’s Hospital, Students with Mental Health Needs, Out-of-Province Children’s Hospital, Local Specialized School for Students with Multiple Disabilities, Local Outdoor Sensory Playground	tour, resources display, conference call and networking, hands-on study-trip


Limited financial resources were a key contextual factor in the implementation of this CoP. Participants from the hosting school division received $25 CAD per year to support their participation, while participants from other school divisions acquired similar funds as needed through divisional budgets or site-based administrative support. This modest financial support enabled attendance but placed limits on travel, honorarium provision, and resource procurement.

### Attendance

Attendance was tracked through meeting logs and participant check-ins. This was a requirement of the school division, not of the facilitators. Participation ranged from consistent core members to rotating attendees. Strategies to promote attendance included flexible scheduling, asynchronous resource sharing, and emphasis on relational accountability.

### Evaluation

As a practice-based implementation, this intervention was informed by descriptive, qualitative data collected throughout its duration via ongoing communication, facilitator reflections, and participant feedback. These data sources supported iterative adjustments to the intervention and provide insight into how the CoP was experienced, delivered, and sustained over time. While formal evaluation was not embedded in the original design of the CoP, the materials and routines used to gather participant feedback over time—such as reflective prompts, meeting summaries, and end-of-year surveys—proved highly useful for evaluating the impact and effectiveness of the intervention. Participants were aware that their ongoing feedback was used to inform and adapt the development of the CoP, which contributed to a shared sense of ownership and responsiveness throughout the intervention. Formal consent was not required for this intervention, as it constituted a quality improvement initiative, and all participants provided implicit consent through their voluntary engagement and awareness that their feedback would be used to inform ongoing planning and development of the CoP.

A multi-modal analysis of implementation feedback was developed post-hoc, leveraging the breadth of available data to retrospectively evaluate the effectiveness, impact, and participant experiences of the CoP over its five-year duration. The analysis design intentionally integrated both deductive and inductive elements, aligning with the intervention’s dual focus on predefined learning objectives and emerging relational and professional outcomes. Data sources included narrative reflections, open-ended participant feedback forms, facilitator notes, and meeting summaries. Additionally, a comprehensive anonymous end-of-intervention feedback form was distributed at the conclusion of year five to support longitudinal reflection and synthesis. Table 3 outlines the scope and distribution of data sources used throughout the five-year implementation of the CoP. The table includes frequency, number of respondents, and roles of contributors for each data type.

**Table 3:** Summary of scope and distribution of data sources used for the Community of Practice activities.


DATA SOURCE	NUMBER COLLECTED	AVERAGE RESPONDENT	COLLECTION FREQUENCY	COLLECTED FROM	NOTES

**Meeting Summaries**	20	8	After each session (4 per year, over 5 years)	Facilitators, Participants	Co-written collaboratively at the end of each session

**Facilitator Notes**	20	2	After each session (4 per year, over 5 years)	Facilitators	Includes planning, reflective, and observational notes

**Open-Ended Feedback Forms**	10	5	Twice annually (after mandatory sessions)	Participants	Collected to assess session impact and suggestions

**Narrative Reflections**	5	5	Annually (after final session each year)	Participants	Participant-generated reflections on learning and impact

**End-of-Intervention Feedback Form**	1	5	End of Year 5	Participants, Facilitators	Anonymous responses from diverse roles and settings


A mixed deductive–inductive thematic analysis was conducted, following the reflexive approach described by Braun and Clarke (2022). The analysis was guided by both predefined learning objectives and an openness to patterns identified through interpretive engagement with the data. A deductive lens was first applied to assess how participant feedback corresponded with the intended learning outcomes of the intervention. Concurrently, an inductive process allowed the researchers to generate additional themes through iterative coding and discussion. Themes such as professional renewal, trauma-informed facilitation, and the emotional toll of working in isolated clinical environments were actively constructed through reflective engagement with the dataset. The two co-facilitators independently reviewed the data and collaboratively developed codes and themes. This reflexive process involved ongoing comparison, refinement of interpretive categories, and attention to how their positionality and shared context within the CoP shaped the analytic process. Discrepancies were discussed to support analytic coherence and ensure that diverse perspectives were considered in the generation of themes.

## Findings

The following findings from the analysis of implementation feedback are presented in two parts: first, themes identified through inductive analysis, followed by findings aligned with the original learning objectives using a deductive approach. As this was a practice-based implementation of a CoP intervention, all findings are drawn from participant-reported experiences and facilitator documentation. The themes reflect interpretive meaning-making within a constructivist framework, informed by the implementation context and educational goals of the CoP.

### Structure and Facilitation

The structure and facilitation of the CoP were praised by participants: “*The role of facilitator and co-facilitator has been unique… it allows for a shared responsibility… Specific topics and areas of need can more readily be accessed and explored resulting in just-in-time learning*.”

Those unable to attend all sessions explained their absences were due to teaching obligations or urgent matters at alternate hospital sites. Others emphasized that they prioritized any session they could attend: “*I would attend any that were available to me as they always had inspiring conversations and well-planned sessions that were able to be immediately applied to my practice as a teacher in hospital*.”

### Contextual and Systemic Challenges

Facilitator notes captured a range of systemic and bureaucratic challenges that influenced the implementation and sustainability of the CoP. These included the difficulty of coordinating meeting times across different school division calendars, limited access to alternative spaces for in-person meetings within hospitals, and challenges advocating for travel time and coverage to support participation in other cities. Facilitators also noted challenges in providing adequate honoraria to guest speakers and knowledge holders given limited financial resources and divisional policies.

### Relational and Professional Impacts

Participants described the CoP as a meaningful and safe space that offered relevant, timely, and context-specific support. Several participants noted the importance of working in a small group environment, which allowed for trust, vulnerability, and deeper conversations: “*The experience of a small group [CoP] has provided a safe environment. This experience would not have been as successful in a large group setting*.” Another participant echoed this, saying, “*Having a more intimate and consistent group over time helps me to develop more meaningful and relevant connections*.”

Participants described how the CoP significantly reduced feelings of being peripheral to broader school division initiatives, fostering a sense of professional inclusion and positioning their specialized roles as integral and valued within their educational communities.

### Relevance to Practice and Identity

Participants also appreciated the real-world applicability of what they learned: “*The importance of understanding and accommodating students with complex medical needs in school settings and shared conversations with hospital and school staff to ensure programming is at its most optimal level for students*.” Others remarked on how the CoP supported their sense of professional purpose: “*Encouragement to continue to focus my learning and attention in a very specific area. Not feeling the need to generalize*.”

Participants appreciated that the CoP was focused directly on their needs in hospital and complex care teaching: “*This [CoP] is focused on a topic that has a direct impact on me and my teaching. I find division-based PD [professional development] often does not relate to my position*.” One participant noted that the PD offered in the CoP was, “*the most practical and valuable experience*” they have had, while another reflected that the CoP gave them a “*heart driven*” and relational lens to apply to curriculum and instruction.

Participants frequently reflected on how the CoP enhanced their sense of professional identity and value, noting that it provided a rare and affirming opportunity to engage deeply with colleagues who share the unique experience of teaching in hospital and complex care settings.

### Alignment with Learning Objectives

Findings that aligned with the predefined learning objectives are summarized below, reflecting participant-reported growth in areas such as trauma-informed instruction, collaboration, and transition planning.

- Participants reported improved confidence and skill in developing trauma-informed and transition-focused educational plans. One participant wrote, “*Has improved my trauma informed practice*.”
- Resource sharing led to the co-development of adaptable toolkits and processes. Another shared the CoP provided, “b*est practices for students with medical and mental health needs… access to practical strategies*.”
- Trust and collaboration grew across sites, breaking down silos between roles and systems. A participant noted their appreciation for “t*he opportunity to connect and share ideas. Guest speakers enhanced our perspectives*.”
- Participants identified the CoP as a space for renewal, describing it as “*a lifeline*” and “*the only place I can talk honestly about this work*.” One participant elaborated, “*I am so grateful to be part of the CoP. It has refueled me professionally*.”
- The small group structure was seen as a strength: “*Smaller numbers are most preferred for me to engage in meaningful conversations*.”
- The CoP seeded further collaborations including cross-site mentorship and shared training initiatives. As noted by one educator, this CoP was “t*he most practical and valuable professional development experience I’ve had*.”

### Knowledge Translation

Knowledge translation efforts extended beyond the CoP meetings themselves. Facilitators and participants shared learnings through a podcast interview, conference presentations, and field trips to other hospital schools to showcase the CoP model and its outcomes (Field & Lewis, 2020; Sickboy Poscast, 2015-present). These knowledge mobilization strategies contributed to increased awareness of the role of hospital school teachers and contributed to the overall impact of the CoP across educational and health system settings.

## Discussion

This CoP intervention demonstrated how collaborative professional learning structures can offer essential professional development for the unique needs of hospital school educators navigating complex and often isolating work environments. The sustained engagement of participants, even as the delivery model shifted due to the COVID-19 pandemic, highlights the relevance and relational strength of this initiative. Participants reported that the CoP created a rare space for interdisciplinary reflection, resource co-creation, and capacity-building centered around the diverse needs of students with medical and mental health conditions. A notable insight was the way the CoP fostered professional identity renewal among educators who often feel peripheral to broader school division initiatives. Through shared storytelling, visual facilitation, and guest knowledge holders, members reconnected with their purpose and each other. The diversity of materials created, ranging from trauma-informed re-entry protocols to student wellness planning tools, illustrates the practical value of the CoP model. While implementation required coordination and iterative adjustment, the resulting community offered significant professional and emotional support to its members. Challenges such as fluctuating engagement and systemic constraints were met with flexibility and care. Critical success factors included distributed leadership, responsiveness to emergent needs, and a trauma-informed lens. One critical recommendation for others seeking to implement a CoP is to integrate evaluation strategies from the onset specifically from members and not just facilitators to school division leadership. Although this CoP yielded rich qualitative feedback, a more robust evaluation framework would support longer-term learning and scalability. Integrating formative and summative assessments throughout the intervention could further refine the model and demonstrate its impact across systems.

Although this paper provides valuable insights into the design and impact of the CoP, it is important to acknowledge the retrospective nature of the analysis. Data were originally gathered to support ongoing facilitation and reflection, rather than formal research. As a result, the findings offer descriptive and context-specific insights but lack the methodological rigor of a prospective design with predefined metrics, limiting causal inference and assessment of effect size. The qualitative approach used here, grounded in participant feedback and facilitator reflection, allowed for depth and contextual richness but presents limitations in terms of generalizability and objective effectiveness assessment. Future studies might strengthen the methodological rigor of similar CoPs by integrating a mixed-methods design. This could include pre- and post-intervention measures, participant-reported outcome tools, or observational fidelity metrics to quantitatively assess changes in practice or student outcomes. These additions would support a more comprehensive understanding of both perceived and measurable impact while maintaining the relational and responsive core of the CoP model. Future directions include formalizing evaluation strategies and extending the model to additional specialized education settings. This intervention contributes to the growing field of practice-based evidence by demonstrating how CoPs can be both effective and sustainable in complex, real-world contexts.

## Conclusion

The Hospital Schools CoP model offers a replicable and responsive approach to professional development that emphasizes collaboration, relational trust, and shared learning which addresses the unique needs of hospital school teachers. By anchoring the intervention in context, values, and community, this CoP helped bridge practice across education and health systems. It demonstrated that with thoughtful facilitation and meaningful engagement, educators working in specialized settings can co-create sustainable professional cultures that center the well-being and success of their students—and of themselves as professionals. While this CoP shows promise, the findings are limited by the small sample size, regional focus, and the retrospective nature of the evaluation. Future development should explore how similar models function across diverse jurisdictions, integrate structured evaluation from the outset, and assess long-term impacts on both teacher practice and student outcomes.

## Additional File

The additional file for this article can be found as follows:

10.5334/cie.165.s1 Appendix A. Community of Practice Planning Guide and Sample Resources.
