# Supplementary material for: Strengthening Professional Collaboration and Expertise: Implementing and Sustaining a Hospital School Community of Practice
Source: Contin Educ. 2025 Jul 9;6(1):91–103. doi: 10.5334/cie.165 (PMC12247828; doi:10.5334/cie.165)
Supplement: Appendix A. — Community of Practice Planning Guide and Sample Resources. [file cie-6-1-165-s1.pdf]

Supplementary Files

Appendix A. Community of Practice Planning Guide and Sample Resources

Sample material illustrating how to structure and support a Community of Practice. It includes planning templates, curated resources, and strategies designed to foster collaboration among educators and professionals working with student patients and children with chronic illness.

COMMUNITY OF PRACTICE
planning guide

TOPIC:	FACILITATORS:
MEETINGS:	TIME:

POSSIBLE MEMBERS

TOPICS OF INTEREST

PEOPLE AND ORGANIZATIONS OF INTEREST

RESOURCES OF INTEREST

© 2021 Field & Lewis

COMMUNITY OF PRACTICE

sample resources

JOURNAL ARTICLES - HANDOUTS

Harden, C., Rea, H, Buchanan-Perry, I., Gee, B., & Johnson, A. (2020). A multidisciplinary educational approach for children with chronic illness: An intervention case study. Continuity in Education, 1(1), 8-21.

Elam, M., Murpy, C., & Irwin, M. (2019). Validity, reliability, and feasibility of the brief school needs inventory: Evaluating educational risk for students with chronic health conditions. Psycho-Oncology, 28, 1483-1489.

Making a Trauma-Informed Self-Care Plan - The Pennsylvania Child Welfare Resource Center - http://www.pacwrc.pitt.edu/Curriculum/313_MngngImpctTrmtcStrssChldWlfrPrfssnl/hndts/HO06_MkngTrmInfrmdSlfCrPln.pdf

Why Every Second Matters - Saskatchewan Advocate - https://www.saskadvocate.ca/sites/default/files/u11/When%20Every%20Second%20Matters.pdf

PEOPLE - ORGANIZATIONS

Parents of Student Patients

Student Patients

Child Life Specialists

Social Workers

Mobile Crisis

Newcomer Centre

Child Advocate

University Professors

Teachers of Student Patients

School-based Counsellors

Schools in your area

Teachers in your area

Pediatric Nurses

Psychiatry Nurses

Learning Support Teachers

Support local family businesses
ex: 21Treats @21treatsyqr

Support organizations which support your student patients
ex: Beads of Courage

PODCASTS - BOOKS - MOVIES

Facilitating the Learning Experience of Children and AYAs During and After Cancer Treatment - https://treatingbloodcancers.org/e25/

Sickboy Podcast - http://sickboypodcast.com/

Bruce Perry - The Child who was Raised as a Dog

KPJR Film - Resilience: Biology of Stress and the Science of Hope

ADVENTURES - ACTIVITIES

Jim Pattison Children's Hospital (before opening) - Saskatoon, Saskatchewan

Dr. Gordon Townsend School - Calgary Public School Division (virtual)

Nanatawihowikamik: Healing Lodge and Wellness Centre - University of Regina

Caregiver Beads - Beads of Courage - http://www.beadsofcourage.org/wp-content/uploads/2019/10/BOC-Program-Summary-2019.pdf

GooseChase App - Team Building Welcome Scavenger Hunt

REFLECTION AND DISCUSSION TOPICS

Attendance Matters: Burning Issues Regarding School Refusal, Re-entry, and On-time Graduation for students with medical needs

Chronic Pain and School Performance

Collaborative Professionals-Who they are and what they do

Common Stress Reactions: A Self-Assessment

Personal, professional highs, lows, worries, and wins since school buildings have been closed due to Covid-19

© 2021 Field & Lewis

COMMUNITY OF PRACTICE *summary*

community of practice

a self-selected, self-directed group of educators with a common concern, interest, or passion who collaborate regularly with a focus on improving and sharing practices and experiences to enhance student achievement

VALUE OF

- **educates** and **connects** people and organizations
- **cultivates** relevant **professional** development and passion
- **empowers** members and validates learning as work

STARTING

- **initiate** and identify a facilitator
- **share vision** and post a summary of topics and goals
- **recruit** members and promote collaboration

BUILDING

- **identify and introduce** members, experiences, areas of expertise
- be **intentional** and celebrate each member and their involvement
- **engage** and empower members, explore all options

FACILITATING

- **invite** and **welcome** members, plan agenda, take attendance
- **collaborate** and **plan** meeting times, short and long term goals
- **prepare** and **equip** with resources, a schedule and platform

© 2021 Field & Lewis

COMMUNITY OF PRACTICE *summary*

CONNECTION

I define connection as the energy that exists between people when they feel seen, heard, and valued; when they can give and receive without judgment; and when they derive sustenance and strength from the relationship.

~Brené Brown

CONSIDERATIONS

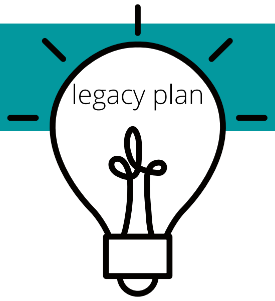

- **utilize** spaces in your area
- **plan** 2-4 meetings/school year
- **determine** size of group based on intent
- **post** highlights in a collaborate space
- **accommodate** needs and trends, topics can change or continue

REFERENCES

E. (2016). Creating Communities of Practice. Retrieved 2021, from http://www.communityofpractice.ca/

R. (2020). Communities of Practice Overview. Retrieved 2021, from https://staff.rbe.sk.ca/cofp

ACTIVE MEMBER TESTIMONIAL

"The three years that I have participated in the Community of Practice facilitated by Heather and Miranda has helped me to more deeply understand the complex psychosocial implications of hospitalization for students. Further, it has highlighted the imperative need for students, families, medical personnel, and school personnel to collaborate while finding solutions to keep students engaged and learning."

~Richie, Hospital Teacher, Inpatient Mental Health, Regina, Saskatchewan, Canada

CONTACT INFORMATION

miranda.field@uregina.ca
heather.lewis@rbe.sk.ca

© 2021 Field & Lewis
